# Consensus-based recommendations for the management of uveitis associated with juvenile idiopathic arthritis: the SHARE initiative

**DOI:** 10.1136/annrheumdis-2018-213131

**Published:** 2018-03-28

**Authors:** Tamas Constantin, Ivan Foeldvari, Jordi Anton, Joke de Boer, Severine Czitrom-Guillaume, Clive Edelsten, Raz Gepstein, Arnd Heiligenhaus, Clarissa A Pilkington, Gabriele Simonini, Yosef Uziel, Sebastian J Vastert, Nico M Wulffraat, Anne-Mieke Haasnoot, Karoline Walscheid, Annamária Pálinkás, Reshma Pattani, Zoltán Györgyi, Richárd Kozma, Victor Boom, Andrea Ponyi, Angelo Ravelli, Athimalaipet V Ramanan

**Affiliations:** 1 2nd Department of Pediatrics, Semmelweis University, Budapest, Hungary; 2 Klinikum Eilbek, Hamburger Zentrum für Kinder- und Jugendrheumatologie, Hamburg, Germany; 3 Department of Pediatric Rheumatology, Hospital Sant Joan de Déu, Universitat de Barcelona, Barcelona, Spain; 4 Department of Ophthalmology, University Hospital Utrecht, Utrecht, The Netherlands; 5 Service de médecine des adolescents, CHU Bicêtre, AP-HP, 78, rue du Général-Leclerc, Paris, France; 6 Department of Ophthalmology, Great Ormond Street Hospital, London, UK; 7 Department of Ophthalmology, Meir Medical Center, Kfar Sava, Israel; 8 Department of Ophthalmology, Uveitis-Center, and Ophtha Lab, at St. Franziskus Hospital, Muenster, Germany; 9 University of Duisburg-Essen, Duisburg, Germany; 10 Department of Rheumatology, Great Ormond Street Hospital, London, UK; 11 Department of Paediatrics, Rheumatology Unit, Anna Meyer Children’s Hospital, University of Florence, Florence, Italy; 12 Department of Paediatrics, Meir Medical Center, Sackler School of Medicine, Tel-Aviv University, Tel Aviv, Israel; 13 Department of Pediatric Rheumatology and Immunology, Wilhelmina Children’s Hospital, University Medical Center Utrecht and University of Utrecht, Utrecht, The Netherlands; 14 Department of Internal Medicine, St. Antonius Hospital, Nieuwegein, The Netherlands; 15 Università degli Studi di Genova and Istituto Giannina Gaslini, Genoa, Italy; 16 University Hospitals Bristol NHS Foundation Trust & Bristol Medical School, University of Bristol, Bristol, UK

**Keywords:** juvenile idiopathic arthritis, Tnf-alpha, methotrexate

## Abstract

**Background:**

In 2012, a European initiative called *S*ingle Hub and Access point for pediatric Rheumatology in Europe (SHARE) was launched to optimise and disseminate diagnostic and management regimens in Europe for children and young adults with rheumatic diseases. Juvenile idiopathic arthritis (JIA) is the most common rheumatic disease in children and uveitis is possibly its most devastating extra-articular manifestation. Evidence-based guidelines are sparse and management is mostly based on physicians’ experience. Consequently, treatment practices differ widely, within and between nations.

**Objectives:**

To provide recommendations for the diagnosis and treatment of JIA-associated uveitis.

**Methods:**

Recommendations were developed by an evidence-informed consensus process using the European League Against Rheumatism standard operating procedures. A committee was constituted, consisting of nine experienced paediatric rheumatologists and three experts in ophthalmology from Europe. Recommendations derived from a validated systematic literature review were evaluated by an Expert Committee and subsequently discussed at two consensus meetings using nominal group techniques. Recommendations were accepted if >80% agreement was reached (including all three ophthalmologists).

**Results:**

In total, 22 recommendations were accepted (with >80% agreement among experts): 3 on diagnosis, 5 on disease activity measurements, 12 on treatment and 2 on future recommendations.

**Conclusions:**

The SHARE initiative aims to identify best practices for treatment of patients suffering from JIA-associated uveitis. Within this remit, recommendations for the diagnosis and treatment of JIA-associated uveitis have been formulated by an evidence-informed consensus process to suggest a standard of care for JIA-associated uveitis patients throughout Europe.

## Introduction

In 2012, Single Hub and Access point for pediatric Rheumatology in Europe (SHARE) was launched with the aim of optimising and disseminating diagnostic and management regimens for children and adolescents with rheumatic diseases. The European League against Rheumatism (EULAR) has produced a number of recommendations in the area of paediatric rheumatology such as juvenile dermatomyositis^1^ using a standardised procedure^2^ and referring to a generalised instrument for guideline assessment: the Appraisal of Guidelines for Research & Evaluation.^3^

Juvenile idiopathic arthritis (JIA) is the most common rheumatic disease in children, and uveitis is the most frequent and potentially most devastating extra-articular manifestation. There are no international consensus statements specifically relating to the diagnosis and treatment of JIA-associated uveitis although there are national guidelines from Germany and Spain.^4 5^ Management is therefore based on physicians’ personal experience; and considerable variation in clinical practice, both in terms of investigation and management of JIA-associated uveitis, was found in an extensive survey of uveitis experts.^6^

With the rapid development of novel therapies for JIA, clear recommendations based on available best evidence and expert opinion (when trial evidence is lacking) will help physicians in the care of patients with JIA-associated uveitis. The uveitis seen with JIA is usually chronic anterior uveitis which is asymptomatic, but acute anterior uveitis can also be seen in the enthesitis-related arthritis subtype.^7 8^ There is a clear need to regularly update those managing patients with JIA uveitis through expert opinion. While the majority of treatments available for the treatment of arthritis have a solid evidence base from clinical trials, corresponding data for patients with uveitis may not be known or the level of evidence may not be as strong at the time of their introduction into clinical practice.

The primary aims of the recommendations forwarded by the Expert Group were to develop agreed strategies to

- prevent or reduce the likelihood of JIA-associated uveitis from occurring and minimise the damage at the time of diagnosis
- recommend treatments and management strategies that would reduce inflammation and prevent the development of those ocular complications most likely to cause irreversible visual loss.

## Methods

A committee of 12 experts (AH, BV, CP, CE, SC-G, IF, JdB, JA, KW, RG, YU, NW) in paediatric rheumatology (n=9) or ophthalmology (n=3) was established to develop recommendations for JIA-associated uveitis based on consensus, but evidence informed, using EULAR standard operating procedures for developing best practice.^2 3^

### Systematic literature search

**Figure 1:**
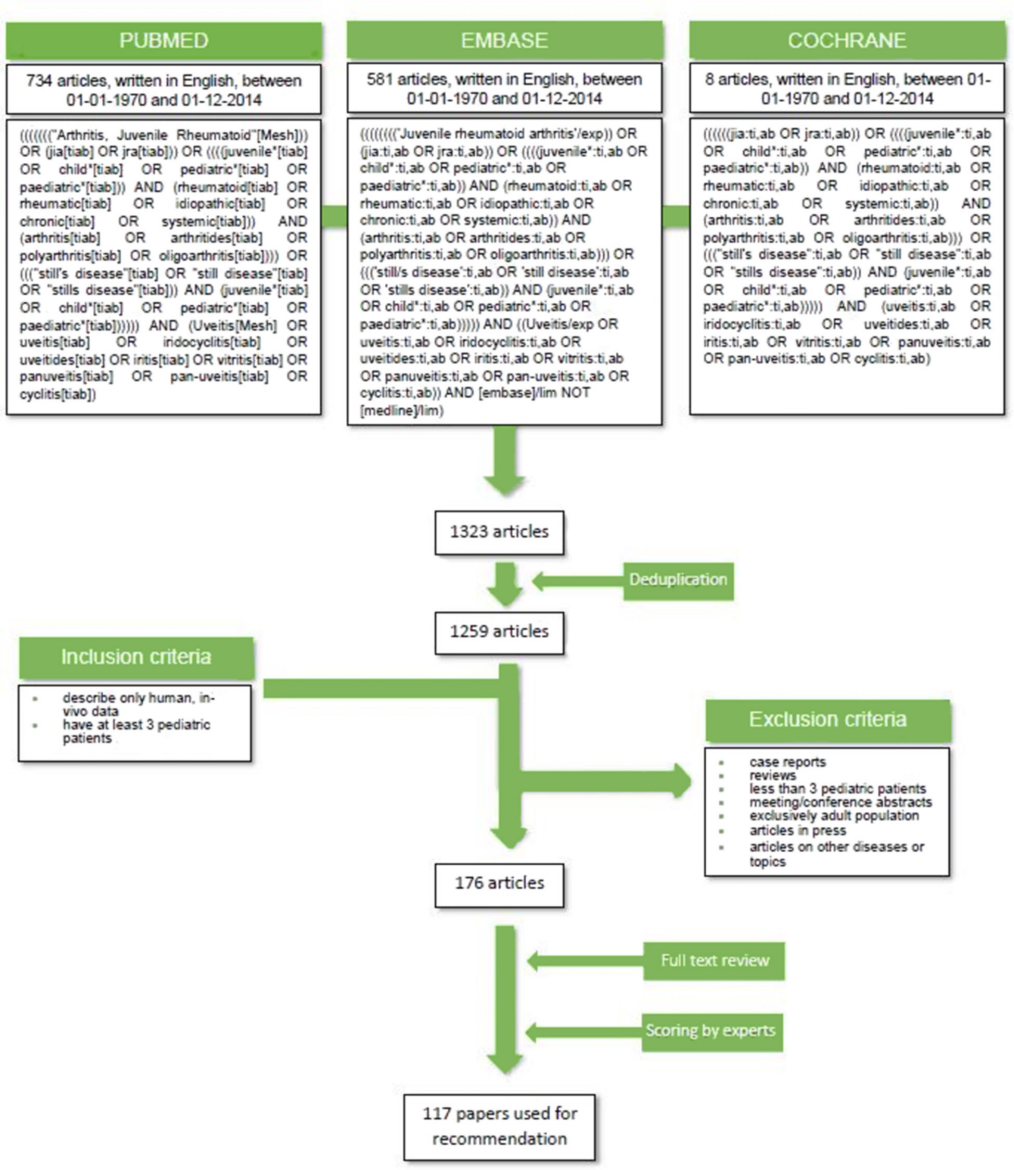
Summary of search strategy for identification of key articles.

The electronic databases PubMed/MEDLINE, Embase and Cochrane were searched independently by two researchers for eligible articles in February 2015. All synonyms of JIA were searched in Medical Subject Headings/Emtree terms, titles and abstracts (figure 1). Reference tracking was performed in all included studies (full search strategy is shown in figure 1). Experts (TC, AP, VB) selected papers relevant to uveitis associated with JIA investigations and/or treatment to be taken forward for validity assessment (inclusion and exclusion criteria shown in figure 1) by members of the expert committee.

### Validity assessment

The selected articles were randomly allocated to the expert group, and two members per paper independently assessed the methodological quality of those papers meeting the inclusion criteria (figure 1). Data were extracted using predefined scoring forms for diagnostic and therapeutic studies. Disagreements were resolved by discussion or by the opinion of a third expert. Adapted classification tables for diagnostic, therapeutic and epidemiological studies were used to determine the level of evidence and strength of each recommendation.^9–11^

### Establishment of recommendations

As part of the EULAR standard operating procedure, experts described the main results and conclusions of each paper, along with their validity and level of evidence. These descriptions were collated by five experts (TC, GS, YU, RG, JdB) and used to formulate provisional recommendations which were reviewed by a panel of three experts (IF, NW, JdB). A summary of the evidence was presented along with each provisional recommendation to the expert committee. The recommendations were revised and discussed at a face-to-face meeting in March 2014 (Genova: 12 participants), using a Nominal Group Technique to reach a consensus.^12^ A non-voting expert (AR) facilitated the process. Recommendations were accepted when ≥80% (10 of 12) of the experts agreed (including all three ophthalmologists).

## Results

### Literature review

The literature search yielded a total of 1323 papers which, after deduplication, left 1259 unique articles. Evaluation of inclusion/exclusion criteria in titles and/or abstracts resulted in a core reference database of 176 articles for which full-text copies were obtained for quality screening by the expert committee. Of these, 117 were selected to support the development of consensus-based recommendations by the expert group (figure 1).

### Recommendations

The sections that follow report the recommendations of the expert committee based on the supporting literature.^13–128^ Tables 1–4 summarise the recommendations, the level of evidence that they provide and the strength of the recommendation, and the percentage of experts who agreed with these assessments.

**Table 1:** Recommendations for diagnosis and screening in juvenile idiopathic arthritis (JIA)-related uveitis

Recommendation	L	S	Agreement (%)	References
1. All patients in whom a diagnosis of JIA is being considered should be screened for uveitis according to a contemporary and audited protocol. Formal screening protocol should be administered in all centres, where patients with JIA are seen.	2A	B	100	13–32
2. Frequency of ophthalmological follow-up visits must be based on disease severity and needs to be decided in conjunction with an expert ophthalmologist.	4	D	100	13–27 33–66
3. Patients with JIA stopping any systemic immunosuppressant are at risk of developing new onset uveitis or recurrence of uveitis after a prolonged remission. After stopping systemic immunosuppression, it is recommended that all patients with JIA are screened by an ophthalmologist at least every three months for at least 1 year.	2B	B	100	67–70

*Agreement* indicates the % of experts that agreed on the recommendation during the final voting round of the consensus meeting.

1A, meta-analysis of cohort studies; 1B, meta-analysis of case–control studies; 2A, cohort studies; 2B, case–control studies; 3, non-comparative descriptive studies; 4, expert opinion; A, based on level 1 evidence; B, based on level 2 or extrapolated from level 1; C, based on level 3 or extrapolated from level 1 or 2; D, based on level 4 or extrapolated from level 3 or 4 expert opinion. L, level of evidence; S, strength of evidence.

**Table 2:** Recommendations for disease activity measurement in juvenile idiopathic arthritis (JIA)-related uveitis

	L	S	Agreement (%)	References
4. There should be good communication between the ophthalmologist and the paediatric rheumatologist concerning changes in disease activity treatment changes and responsibility for treatment monitoring.	3	C	100	71
5. There is a need to develop shared outcome measures to help guide decisions on systemic treatment.	4	D	100	
6. At present, there is no validated biomarker to follow the activity of uveitis.	2A	B	100	4 21 29 31 32 37 41 46 72–76
7. At present, no widely accepted definition of inactive disease for JIA-related uveitis is available. The goal of treating JIA-associated uveitis should be no cells in the anterior chamber. The presence of macular and/or disk oedema, ocular hypotony and rubeosis iridis may require anti-inflammatory treatment even in the absence of AC cells.	2B	B	100	4 69 78
8. We recommend 2 years of inactive disease off topical steroids before reducing systemic immunosuppression (both DMARDs and biological therapies).	3	C	92	67

*Agreement* indicates the % of experts that agreed on the recommendation during the final voting round of the consensus meeting.

1A, meta-analysis of cohort studies; 1B, meta-analysis of case–control studies; 2A, cohort studies; 2B, case–control studies; 3, non-comparative descriptive studies; 4, expert opinion; A, based on level 1 evidence; B, based on level 2 or extrapolated from level 1; C, based on level 3 or extrapolated from level 1 or 2; D, based on level 4 or extrapolated from level 3 or 4 expert opinion. DMARD, disease-modifying anti rheumatic drugs; L, level of evidence; S, strength of evidence.

**Table 3:** Recommendations for treatment in juvenile idiopathic arthritis (JIA)-related uveitis

	L	S	Agreement (%)	References
9. Active uveitis in JIA usually requires immediate treatment.	2B	B	100	69 71 78–80
10. Topical corticosteroids (preferably prednisolone acetate or dexamethasone) are the first-line treatment of anterior uveitis.	4	D	100	81
11. Topical and systemic NSAIDs have no demonstrable effect as monotherapy, but may be used as additional therapy.	3	C	92	79 81 82
12. Systemic immunosuppression in active uveitis is recommended if poor prognostic factors are present at the first visit. Poor prognostic factors including lack of remission later on during the disease course require systemic immunosuppression.	2A		100	4 19 22 29 55 56 65 78 83 84
13. Systemic immunosuppression is recommended if inactivity could not be reached within 3 months or inflammation is reactivating during steroid dose reduction.	2B	B	100	55 59 68 69 78 80 85–87
14. Methotrexate is the first choice as systemic immunosuppression.	4	D	100	68 84 88–95
15. In case of methotrexate inefficacy or intolerance, adding or switching to biological treatment is recommended.	3	C	92	91–104
16. The use of anti-TNF treatment strategies *(adalimumab>infliximab>golimumab)* is recommended in patients with uveitis refractory/resistant to DMARD therapy, principally methotrexate.	3	C	100	86 100 101 104–117 120–124 126 127
17. Based on the current evidence, etanercept should not be considered for JIA-associated uveitis.	1B	A	100	87 100 109 117–121
18. Switching between different anti-TNF treatments might be valuable if uveitis is refractory to the first anti-TNF, even though the present evidence comes from small case series or inception cohorts.	3	C	100	87 113 116 122
19. In case of lack of efficacy, consider testing for antidrug antibodies and drug trough level. If the patient has no antibodies but has low trough levels, consider increasing the dose or shortening the interval.	4	D	100	
20. Tocilizumab, rituximab and abatacept might be potential options for cases refractory to previous anti-TNF therapy.	3	C	100	123–125

*Agreement* indicates the % of experts that agreed on the recommendation during the final voting round of the consensus meeting.

1A, meta-analysis of cohort studies; 1B, meta-analysis of case–control studies; 2A, controlled study without randomisation; 2B, quasi-experimental study; 3, descriptive study; 4, expert opinion; A, based on level 1 evidence; B, based on level 2 or extrapolated from level 1; C, based on level 3 or extrapolated from level 1 or 2; D, based on level 4 or extrapolated from level 3 or 4 expert opinion.; DMARD, disease-modifying antirheumatic drugs; L, level of evidence; NSAIDs, non-steroidal anti-inflammatory drugs; S, strength of evidence; TNF, tumour necrosis factor.

**Table 4:** Recommendations for future plans in juvenile idiopathic arthritis (JIA)-related uveitis

	L	S	Agreement (%)	References
21. Validated outcome measures for JIA-associated uveitis are needed	3	C	100	73 126–128
22. Controlled clinical trials are needed for JIA-associated uveitis	1B	A	100	119

*Agreement* indicates the % of experts that agreed on the recommendation during the final voting round of the consensus meeting.

1A, meta-analysis of cohort studies; 1B, meta-analysis of case–control studies; 2A, cohort studies; 2B, case–control studies; 3, non-comparative descriptive studies; 4, expert opinion (diagnostic studies); 1A, meta-analysis of randomised controlled trials; 1B, randomised controlled study; 2A, controlled study without randomisation; 2B, quasi-experimental study; 3, descriptive study; 4, expert opinion (therapeutic studies); A, based on level 1 evidence; B, based on level 2 or extrapolated from level 1; C, based on level 3 or extrapolated from level 1 or 2; D, based on level 4 or extrapolated from level 3 or 4 expert opinion, level of evidence; S, strength of evidence.

### Background

JIA is the most common chronic rheumatic disease in children with an incidence of 8.2 (7.5–9.0)/100 000 of the population under 16 and an annual prevalence of approximately 70.2 [16 – 140]/100 000.^129 130^ The wide prevalence range has been attributed to the different study designs employed, but the incidence is thought to vary little worldwide.^130^ The incidence of JIA-associated uveitis is thought to be approximately 1/10 000 and there is some evidence that it is less frequent in oriental populations with JIA.^7 131^

Structural complications, some of them leading to irreversible visual loss, include cataracts, glaucoma, band keratopathy, macular oedema, retinal detachment and sequelae associated with chronic hypotony. The uveitis usually has an insidious time course and can be chronic or recurrent but, most frequently, JIA-associated uveitis is a chronic relapsing condition lasting several years. It almost universally starts as an anterior uveitis, but in rare instances can become a panuveitis. JIA-associated uveitis is usually asymptomatic in the age group in which it most commonly develops (age 3–7 years), but severe inflammation may cause symptomatic pain and redness and these symptoms and signs, as well as pupil distortion, may be noticed by carers and lead to early referral. Reduced visual function is an uncommon cause of presentation in small children unless it is severe and usually secondary to irreversible structural damage. The presence of antinuclear antibodies (ANA), oligoarthritis and early onset of arthritis are predominant risk factors for chronic anterior uveitis in those with JIA, and improved identification of children at risk is a key priority for targeted screening programmes. This exercise only looked at chronic anterior uveitis associated with JIA and not at acute anterior uveitis seen in human leukocyte antigen (HLA) B27-positive children with enthesitis-related arthritis.

Timely and aggressive treatment is clearly needed where there is significant damage at the time of diagnosis. However, the variable course of the disease means that some significant events may occur years after therapy commences and there remains considerable disagreement among expert practitioners about the timing and indications for treatment escalation.^132 133^

### Recommendations for diagnosis and screening in JIA-related uveitis

#### Screening for JIA-associated uveitis

All patients with JIA should be screened for uveitis according to contemporary and audited screening protocols which should be implemented in all centres in which patients with JIA are being managed.^42 43^ There is no necessity for the screening process to take place in the same institution as the rheumatological care. It is especially important, however, that all children in whom the diagnosis of JIA is being considered should have a timely check by a local ophthalmologist rather than wait for confirmation of the diagnosis from a paediatric rheumatology referral centre. It is the responsibility of paediatric rheumatologist to ensure children with JIA are referred for screening. Screening starts with all individuals with a ‘suspected’ diagnosis of JIA and the clinical responsibility for organising this service needs to be clear. This is independent of whether it is the remit of the ophthalmologist not specialised in uveitis, paediatrician or rheumatologist (table 1). The evidence published to suggest that the risk factors originally proposed for the development of chronic uveitis in the JIA population (early-onset arthritis, ANA positivity and oligoarticular subtype) is suboptimal: the expert group noted that despite the fact that a number of screening protocols have been published there is no evidence to suggest that any one of them is superior.^5 23 26 61 74^

Advances in genetics have not found any more specific diagnostic markers than the HLA types originally reported as being associated with both oligoarticular disease and uveitis.^14 15 30 32 38 48 50 56^ There is no evidence, at present, that genotyping adds specificity to the established clinical risk factors on which contemporary screening programmes are based.

Despite the advances in subtyping ANAs in other rheumatic disorders, there has been little advance in our understanding of the association of ANAs with the risk of JIA-associated uveitis^28 34 35 41 60 67^ although antibodies to nuclear structures such as histones and chromatin^16 21 41 52–54 64 77^ and ocular antigens have been reported.^13 33 51 75^ Two studies found that children with higher inflammatory activity (as determined by higher erythrocyte sedimentation rate (ESR) values) when oligoarthritis or polyarthritis was diagnosed had an increased risk of developing uveitis.^27 31^

There is a clear unmet need to adapt screening policies to the contemporary usage of early systemic immunosuppressant treatment of arthritis^57 61 62 64^ and novel biomarkers and future genotyping may improve targeting of the population being screened.

#### Monitoring during follow-up

The risk of visual symptoms and potential for relapse in patients initially responding to treatment highlights the necessity for maintained regular close ophthalmological scrutiny. The expert group recommended that the frequency of ophthalmological follow-up should be based on ocular disease severity and decided upon in conjunction with an expert ophthalmologist.

#### Screening after stopping treatment of uveitis

Methotrexate (MTX) is the immunosuppressive therapy of choice in patients with JIA-related uveitis (see recommendation 14, table 3). Once long-lasting remission of uveitis is achieved, MTX is usually stopped, but the optimal period of disease control prior to withdrawal of both topical and systemic treatment remains unclear. Patients stopping MTX are at risk of developing new onset uveitis or recurrence of uveitis after prolonged remission in the first year. Indeed, the majority of patients in a recently reported series relapsed within 24 months of stopping therapy.^67–70^ Consequently, after stopping immunosuppression with MTX (prescribed for arthritis or uveitis) it is recommended that all patients with JIA are screened by an ophthalmologist at least every three months for a minimum of 1 year.

It also remains unclear for those patients on multiple treatments, in which order treatments are best withdrawn. Relapse of uveitis after withdrawal of MTX appears to be delayed in older patients, those who have been on treatment for longer duration.^67^ The authors recommended that the period of uveitis inactivity should be >2 years before MTX is withdrawn.^67^ The expert group also recommend that patients should have 2 years of inactive disease while not using topical steroids before reducing systemic immunosuppression.

There is a clear need for continuing monitoring in the early period of remission after medications are stopped, especially in patients on long-term therapy, topical or systemic, which had maintained disease control. The expert group recommends that monitoring of disease in remission by an ophthalmologist should be at least every three months and should continue for at least 3 years off all forms of treatment. The length of remission best predicting lifetime remission remains unknown. More robust data on effective screening strategies are required.

### Recommendations for disease activity measurement in JIA-associated uveitis

A pivotal goal in the management of JIA-associated uveitis is to minimise loss of vision through the early diagnosis of ocular morbidity. Early referral and appropriate treatment to eliminate ocular inflammation are seen to be crucial in preserving visual acuity. Visual loss is mainly caused by glaucoma, macular damage from inflammatory oedema, hypotony or amblyopia: structural damage can occur prior to diagnosis and also arise following years of poorly controlled inflammation.

Multiple studies have found delayed presentation with damage at diagnosis, surgery and length of follow-up to be the major risks for lifelong visual loss.^4 68 78^ Male gender and non-Caucasian race may be additional risk factors for some complications.^18 19 37 56 65 71^ Immunosuppressive treatment is therefore aimed at reducing agreed measures of active intraocular inflammation which include the level of cellular infiltrate, breakdown of vascular barriers (eg, flare) and macular oedema: few of these measures have been validated and different markers may be appropriate in eyes with different levels of structural damage or at different stages of disease.^134^

In one study, an increase in anterior chamber cell grade was associated with elevated rates of visual loss in a dose-dependent fashion, whereas immunotherapy was associated with a reduced risk of visual loss, particularly for the 20/50 or worse outcome (HR 0.40; P<0.01).^78^ Others have found anterior chamber flare a better predictor of visual loss.^63^

There are no established biomarkers currently available for predicting disease activity or guiding treatment in JIA-associated uveitis and research efforts in this area need to be intensified (table 2).^24 32 34 35 40 44 49 75–80^

In a small group of children with JIA-related anterior uveitis, serum interleukin-2R levels were significantly increased,^72^ while in a larger cohort of children there was a significant correlation between the presence of anterior uveitis and aqueous humour levels of transthyretin.^73^ Until reliable biomarkers are found, management relies on frequent ophthalmic examination. Future opportunities might include gene expression and proteomic profiling of the serum, peripheral blood leucocytes and aqueous humour; measurement of acute-phase reactants; HLA typing and determination of ANAs.^74^ Evidence-based guidelines by the German Ophthalmological Society, the Society for Childhood and Adolescent Rheumatology and the German Society for Rheumatology noted that macular oedema, ocular hypotony and rubeosis iridis are often associated with chronic inflammation and anti-inflammatory treatment should be initiated (or intensified) even in the absence of cells in the anterior chamber.^4^ The expert group recommended that the goal of treating JIA-associated uveitis should be no cells in the anterior chamber although this may not be practically possible (table 2). With reference to the Standardization of Uveitis Nomenclature (SUN) Working Group, a multinational interdisciplinary group (MIWGUC) developed a set of core outcome measures for uveitis that may provide a framework for evaluating disease severity and its course, risk for structural impairment, levels of impairment in visual function and responses to treatment.^74^ These would be invaluable in clinical studies involving patients with JIA-associated uveitis. However, these proposed outcome measures remain to be validated in children.^135^ To best guide treatment decisions, there should be good communication between the ophthalmologist and the paediatric rheumatologist concerning changes in disease activity treatment changes and responsibility for treatment monitoring. Recently published guidance for management of non-infectious uveitis in adults has some important principles for the management of panuveitis in all age groups.^136^

### Recommendations for treatment in JIA-associated uveitis

Active uveitis in JIA usually requires immediate treatment. In a comparison of two cohorts of patients with new-onset JIA, separated by a 10-year interval, the more recently treated patients received early intensive treatment and close monitoring, and reported fewer complications and milder uveitis with visual loss avoided in most cases.^80^ Treatment factors reported to be associated with improved outcomes included introduction of immunosuppressive therapy earlier in the course of the disease or at a younger age^69^ and treatment with immunosuppressants generally.^71 78^

Based on past usage, the expert group recommend that topical corticosteroids (preferably prednisolone or dexamethasone) are the first-line treatments of choice for both acute and chronic anterior uveitis.^4 5 81 129^ Children with JIA-related uveitis are frequently treated with topical corticosteroids over extended periods, and this increases the risk of cataract formation and glaucoma.

One study found that the increased risk of cataract formation with high-dose topical steroids was independent of active uveitis or presence of posterior synechiae.^81^ The risk increased as the number of drops of topical corticosteroids instilled increased. The data suggested that patients may be treated with low doses of topical corticosteroids (≤3 drops daily) over moderate periods of follow-up (median 4 years; range 0.5–15 years) with a low risk of developing cataracts.^81^ Among eyes receiving ≤2 drops daily, the incidence of cataract was 0/eye year. There appears to be no evidence to suggest that less potent topical steroids reduce adverse effects in this patient population (table 3). Systemic corticosteroids are not usually preferred in children due to risks of growth suppression and osteopenia; however, they are potentially helpful in individual cases for rapid control of severe uveitis or in the presence of macular oedema.

In a retrospective study, the adjunctive use of non-steroidal anti-inflammatory drugs (NSAIDs) for the treatment of chronic iridocyclitis was evaluated in 14 patients, 8 with JIA and 6 with idiopathic iridocyclitis.^82^ In all patients, the activity of the iridocyclitis improved with the addition of NSAIDs to their treatment regimens, permitting reduction in the dose of corticosteroid drugs. These data suggest that NSAID therapy may have an adjunctive role in the treatment of chronic iridocyclitis in childhood, but not as monotherapy.

Systemic immunosuppression for active uveitis is recommended to reduce complications in those cases where topical steroids are insufficient to eliminate ocular inflammation short term, or such high doses are required that treatment risks outweigh the beneficial effects. However, the threshold for introducing systemic treatment is lower in those with multiple risks for visual loss as discussed earlier.^129^ Immediate systemic immunosuppression in active uveitis is recommended if poor prognostic factors are present at the first visit. Studies suggest instituting aggressive immunosuppression in high-risk patients even before there is any clear evidence of complications developing.^27 39^ Poor prognostic factors include uveitis antedating arthritis^19 22 74^; posterior synechia^55 68 74 75^; male gender^19 22 55^; band keratopathy, glaucoma and cataract^55^; poor initial vision, hypotony, macular oedema and dense vitreous body opacification^55^; and lack of remission later on during the disease course (table 3). Age at the time of uveitis onset does not appear to be a risk factor.^22 56^

### Definition of treatment failure

Treatment failure should lead to a change in treatment dose, route or modality taking into consideration the fact that many of the drugs require different times to achieve their optimal effect and there are sometimes only a limited number of drugs available. Inappropriate drug changes may result from difficulties in achieving consensus on the definition of treatment failure. In a retrospective study, the clinical outcome of 23 patients with JIA-associated uveitis unresponsive to corticosteroids was ascertained. Uveitis was controlled using immunosuppressive therapy in all cases.^86^ Patients treated within 4–30 months from onset of uveitis achieved better improvement of vision compared with patients who received immunosuppressive therapy after 3 years (P<0.005 for right and left eyes pooled; P=0.0075 for best eyes; P=0.0375 for worst eyes). These data are supported by findings from the SITE study which reported that among patients with JIA-associated uveitis receiving tertiary care use of immunosuppressants reduced the risk of vision loss by about 60%.^78^ The expert group recommended that systemic immunosuppression should be used if inactivity cannot be achieved with topical steroids within 3 months or inflammation is reactivated during steroid dose reduction. The benefits of immunosuppressive therapy are now well recognised, and, in general, patients in remission received this treatment earlier in the course of disease compared with patients who relapsed.^55 59 68 78–87^

Based on findings of efficacy and tolerability/safety from a number of studies, the expert group recommended that MTX is the immunosuppressive therapy of choice in patients with JIA-related uveitis.^68 84 88–90^ Other forms of immunosuppressive therapy such as azathioprine, sulfasalazine, mycophenolate mofetil, cyclosporine and leflunomide have been assessed in patients with uveitis, often in MTX-resistant cases.^91 95^ The studies have generally included a small numbers of patients, and in the case of cyclosporine, the clinical efficacy was poor and the authors noted that it has limited value in this indication.^94^ The results with other forms of immunosuppressive therapy were more encouraging, but were in fewer patients.^91–93 95^ At this stage they may prove to be useful treatment options in patients not responding to, or who cannot tolerate, MTX and they also have a place accompanying biological treatment in those who are MTX-intolerant. There is also evidence of the greater effectiveness of MTX in controlling arthritis in JIA compared with other conventional immunosuppressants. It is not uncommon for treatment to fail when treatments other than MTX are used because of recurrent arthritis rather than failure to control the uveitis.

As noted above, the evidence with other forms of immunosuppressive therapy in patients with JIA-related uveitis is low,^91–95^ whereas many conventional immunosuppressants are used in adult onset uveitis with no specific agent favoured as the first choice. In both adult and childhood patients who fail to respond to conventional immunosuppressants, expert groups recommend adding or switching to biological treatments. There is a growing number of studies supporting the use of biological therapy in MTX refractory uveitis.^93–108^ A wide range of biological therapies have been investigated in JIA-related uveitis refractory to conventional therapy, with adalimumab being the most widely studied; other agents investigated include infliximab, daclizumab, etanercept, golimumab, abatacept, tocilizumab and rituximab.^96–108 123–125^

All of the biological therapies investigated produced some benefit in patients with uveitis refractory to conventional therapy.^86 100–127^ There are very few comparative studies. One study found infliximab to be significantly superior to etanercept in children with refractory JIA-associated chronic uveitis.^100^ In another small study, adalimumab was more efficacious than infliximab when used as first-line anti-TNF treatment.^106^ Adalimumab also produced higher remission rates versus infliximab in the medium-term treatment (at least 12 months) of patients with JIA-related uveitis^107^ and in children with uveitis it was significantly superior to infliximab in maintaining remission over a 3-year treatment period.^102^ The expert group recommends treatment with anti-tumour necrosis factor (TNF) agents (adalimumab>infliximab>golimumab) in patients with uveitis refractory/resistant to disease-modifying antirheumatic drug (DMARD) therapy, principally methotrexate.^86 100–127^

The use of TNF inhibitors in uveitis was hypothesised based on their proven efficacy in a range of systemic inflammatory disorders including JIA, rheumatoid arthritis and Crohn’s disease.^109^ Etanercept is a recombinant DNA dimeric fusion protein that antagonises TNF-α, and it has proven to be effective in children with polyarticular JIA.^118^ Evidence for the clinical benefit of etanercept in uveitis has generally been disappointing; it was associated with a high relapse rate and a high risk for developing uveitis flares.^87 100 109 117–121^ Based on these findings, the expert group recommend that etanercept should not be considered for JIA-associated uveitis.

Findings from studies including small numbers of patients provide evidence that if treatment with one anti-TNF agent becomes ineffective switching to a different anti-TNF agent could prove to be clinically beneficial.^87 113 116 122^ The efficacy and safety of adalimumab was evaluated in in 26 children with JIA resistant to current therapy (disease-modifying drugs in 17 cases and anti-TNF agents in 9 cases). Switching to adalimumab had a beneficial impact on disease control in 17 (65.4%) of patients.^113^ In total, 17 patients with severe recalcitrant uveitis (resistant to etanercept, infliximab, adalimumab, rituximab or abatacept) were switched to golimumab and 14 achieved a positive response, and in 12 of these the disease was inactive at the final visit (mean duration 22 months; range 6–29 months).^116^ Dhingra *et al* reported preliminary evidence that in seven cases of refractory uveitis switching between biological agents (over a period of 5–24 months) helped to control intraocular inflammation.^122^

While the literature search revealed no direct evidence of the effects of low drug trough levels or the development of anti-drug antibodies (ADAs) on the clinical efficacy of biological agents in patients with uveitis, the expert group considered findings from other clinical settings. They concluded that in cases of loss of efficacy over time consideration should be given to testing for ADAs and drug trough levels.^137 138^ If the patient has no antibodies, but has low trough levels, increasing the dose or shortening the interval may be an option.^139^

In keeping with an earlier recommendation (18, table 3), there are data from small studies that in patients with JIA-related uveitis refractory to conventional therapy and at least one anti-TNF therapy switching to drugs such as abatacept, rituximab or tocilizumab may be beneficial.^123–125^ This included patients in whom the main cause of poor visual acuity was macular oedema.^125^ There is now growing evidence for the role of tocilizumab in macular oedema associated with uveitis.^140 141^ There is also an ongoing trial of tocilizumab in children with anti-TNF refractory JIA-associated uveitis (http://www.aptitude-trial.org.uk/).

The optimum time for surgery in children with complications from refractory uveitis has not been addressed as a recommendation due to paucity of evidence. Recent literature does demonstrate that a significant number of children with uveitis still require surgery for complications.^142^

### Recommendations for future plans in JIA-related uveitis

A MIWGUC identified the need for clinical trials and longitudinal studies to determine the benefits and costs of health interventions in this setting.^74^ To achieve this the group proposed a core set of outcomes aimed at ensuring that changes in relevant outcomes were measured, and that standardisation of outcome measures would facilitate data pooling and comparisons between interventions (tables 4 and 5). The outcomes should be agreed on by both researchers and patients, and they will provide a common focus for interventional studies. Disease-specific and universally agreed on validated outcomes are likely to reduce selective reporting and reporting bias.^74^ A limitation of this core set of outcomes is that, despite the fact that there was consensus by the Working Group regarding their utility, they still remain unvalidated. Visual impairment has a significant impact on the quality of life (QOL) of patients with JIA-related uveitis and vision-related QOL relates to the degree to which vision impacts the individual’s ability to perform activities of daily living as well as social, emotional and economic well-being.^126–128^ The Effects of Youngsters’ Eyesight QOL is a useful instrument for measuring the effects of uveitis on QOL which is currently being validated.

**Table 5:** Proposed domains and items for outcome measures of juvenile idiopathic arthritis (JIA)-associated uveitis (from the Multinational Working Group in JIA-related uveitis)^74^

Domains	Items
Grade of cells in anterior chamber	Slit-lamp examination (according to SUN criteria)
Grade of flare in anterior chamber*	Slit-lamp examination for routine clinical practice and prospective trials (according to SUN criteria) Laser flare photometry for prospective trials
Number of visits with active uveitis	Records of treating physician Duration of activity over a minimum of four visits/year
Visual acuity (appropriate test for age)	Best-corrected visual acuity Thresholds: ≤20/50, ≤20/200 and no light perception Estimate contribution of amblyopia, yes/no
Development of structural complications	Synechiae, yes/no Initial and additional Ocular hypotony, yes/no (<5 mm Hg) Ocular hypertension, yes/no (>21 mm Hg) Glaucoma, yes/no Cataract, yes/no Band keratopathy in the central cornea, yes/no Macular oedema by optical coherence tomography, yes/no Funduscopy and optical coherence tomography for routine clinical practice (for macula and optic disc) Funduscopy and optical coherence tomography for prospective trials Epiretinal membrane formation, yes/no Funduscopy for routine clinical practice Funduscopy and optical coherence tomography for prospective trials
Quality of life	Childhood Health Assessment Questionnaire Child Health Questionnaire Pediatric Quality of Life Inventory Uveitis-specific quality of life instrument (not yet available for non-English speaking countries)
Overall uveitis-related disability	Assessment by parents, visual analogue scale Assessment by children, visual analogue scale Assessment by treating ophthalmologist, visual analogue scale Assessment by treating paediatric rheumatologist, visual analogue scale
Social outcome	School/kindergarten absence
Anti-inflammatory medication*	Reduction of corticosteroid dose—topical dose—systemic dose
Surgery*	Yes/no
Biomarkers	Research tools (not currently available

*Not an outcome measure, but should be documented.

SUN, Standardization of Uveitis Nomenclature.

The expert group indicated a need for more well-controlled clinical trials in children with JIA-related uveitis to provide the scientific best evidence in the areas of diagnosis, screening, disease activity and treatment to enable the optimal care of these patients.

## Discussion

Following a systematic review of the literature and Nominal Group Technique methodology, under the auspices of SHARE and EULAR operating procedures, 22 recommendations for the screening, diagnosis, disease activity monitoring, treatment and future plans for children with JIA-associated uveitis were accepted with at least 80% agreement. In a disease setting where the evidence base is limited by small numbers of patients, and which is developing rapidly, these expert recommendations should help specialists with the evidence-based advice to provide optimal care for their patients.

It should be noted that, in general, the level of evidence was quite low with 13 of 22 recommendations being level 3 or 4, seven level 2 and only two level 1. This highlights the need for more research in this clinical setting where a number of new therapies, particularly biological agents, have been introduced in recent years. At the time that the data search for this article took place, the expert group noted a need for more well-controlled clinical trials in children with JIA-related uveitis. The goal being to ensure that scientific best evidence is used to support optimal treatment. In the interim period, but clearly outside the search time frame of this review, a well-designed randomised controlled trial (RCT) has been published which compared adalimumab with placebo in children with JIA-related uveitis who were taking a stable dose of MTX.^135^ Active treatment was shown to control inflammation and was associated with a lower rate of treatment failure compared with placebo. These results do not alter the recommendations of the expert group, but reinforce the intent of recommendation 15 in table 3: ‘In case of methotrexate inefficacy or intolerance, adding or switching to biological treatment is recommended’. The only difference being that this recommendation is now supported by level 1 evidence, rather than level 3 evidence. More recently, another smaller RCT of adalimumab has also been published showing efficacy of adalimumab in JIA–uveitis although this study used flare as the primary outcome measure.^143^ The utility of biological therapies is receiving wider attention, for example, a number of studies have reported the benefits of tocilizumab.^141 144^ These findings from outside the systematic data search time period emphasise the need to regularly update the recommendations of the JIA-associated uveitis expert group so as to provide the highest levels of care in this clinical setting.
